# GS-Impute: A neural network framework for accurate imputation of low-density markers in across-population genomic selection

**DOI:** 10.1016/j.xplc.2026.101821

**Published:** 2026-03-10

**Authors:** Xin Wang, Zhenting Jiang, Tongtong Ding, Ying Cao, Kai Zhou, Guangning Yu, Pengcheng Li, Zefeng Yang, Xuecai Zhang, Shizhong Xu, Yang Xu, Chenwu Xu

**Affiliations:** 1 College of Information Engineering, Yangzhou University, China/Jiangsu Engineering Research Center for Knowledge Management and Intelligent Service, Yangzhou, Jiangsu 225009, China; 2 Key Laboratory of Plant Functional Genomics of the Ministry of Education/Jiangsu Key Laboratory of Crop Genomics and Molecular Breeding/Jiangsu Co-Innovation Center for Modern Production Technology of Grain Crops, College of Agriculture, Yangzhou University, Yangzhou, Jiangsu 225009, China; 3 Nantong Institute of Technology, Nantong, China; 4 International Maize and Wheat Improvement Centre (CIMMYT), Texcoco, Mexico; 5 Department of Botany and Plant Sciences, University of California, Riverside, Riverside, CA, USA

**Keywords:** genotype imputation, deep learning, neural network, genomic selection, across-population

## Abstract

Genomic selection (GS) holds great promise for accelerating breeding progress in plants, and the advancement of across-population GS is essential to realize its full potential. However, conventional across-population GS heavily relies on precisely aligned markers across dense genotypes, whereas the feasibility of using flexible low-density markers remains underexplored. This study developed GS-Impute, a residual convolutional denoising autoencoder-based neural network framework that enables accurate genotype imputation for low-density across-population GS. A key breakthrough of GS-Impute is an automatic matching algorithm that resolves the persistent challenge of targeted training in the presence of both sporadic and systematic missing data. Additionally, GS-Impute incorporates a data augmentation strategy and several advanced techniques to enhance imputation accuracy, including residual blocks, dynamic learning-rate optimization, and layer normalization. Comprehensive evaluations across rice and maize breeding populations demonstrated that GS-Impute outperforms the latest versions of established benchmark tools, including Beagle5.4, Minimac4, and STICI. Importantly, the results indicate that GS-Impute makes across-population GS feasible with low-density markers, establishing a resource-efficient strategy with the potential to transform genomic breeding programs.

## Teaser

This study introduces GS-Impute, a neural network framework developed for accurate imputation of low-density markers in across-population genomic selection. Comprehensive evaluations in rice and maize show that GS-Impute outperforms established benchmark tools, enabling low-density genomic selection to serve as a resource-efficient strategy for breeding programs.

## Introduction

Genomic selection (GS) utilizes genome-wide markers to estimate breeding values, enabling more comprehensive and reliable selection (Meuwissen et al., 2001; Wang et al., 2018). This approach holds substantial promise for reducing costs (Hickey et al., 2017; Danguy des Déserts et al., 2023) and accelerating crop breeding progress in the era of Breeding 4.0 (Wallace et al., 2018). However, current GS research predominantly relies on cross-validation methodologies, in which a single population is partitioned into training and validation sets to assess model performance. Although this approach proves effective for comparing the predictability of different statistical models, it primarily reflects selection benefits within a single population. Consequently, these methods fail to evaluate the predictability of across-population GS (Haile et al., 2021), which is essential for practical implementation in crop breeding programs. The conventional approach to across-population GS fundamentally relies on the assumption of uniform marker positions across diverse populations. To date, limited research has examined GS across populations in crops, and existing studies are based either on shared marker positions from identical gene chips (Haile et al., 2021) or on aligned markers derived from dense genotypes (Wang et al., 2017; Sarinelli et al., 2019). However, the widespread adoption of these approaches faces considerable challenges due to the high costs associated with sequencing and the inherent variability among sequencing platforms. Therefore, the development and application of cost-effective low-density chip-based strategies for across-population GS is a critical requirement. Genotyping by sequencing represents an attractive and low-cost alternative to SNP arrays (Davey et al., 2011; Gorjanc et al., 2017); it has been widely used to obtain SNP markers in GS studies (Xu et al., 2021). Under the more general condition of low-density markers, relatively few studies have investigated schemes for across-population GS in crops. The inference of ungenotyped markers is regarded as genotype imputation, in which reference-panel-based methods can be used to standardize marker positions across multiple populations. Pedigree relationships among most crop breeding materials are often unclear. Unrelated individuals typically do not share long haplotypes, but shorter regions can still be identical by descent because of the presence of common ancestors (Treccani et al., 2023). Although genotype imputation for crop GS is theoretically feasible, the effectiveness of across-population GS with low-density markers remains uncertain due to limited experimental validation.

In the past decade, several methods for genotype imputation have been developed and can be classified into two categories. The first category includes imputation methods such as the k-nearest neighbor (KNN) algorithm, singular value decomposition, and certain classification and regression algorithms, which do not require haplotype information from a reference population (Song et al., 2020). These methods can be used to infer missing genotypes and thus improve the power of genome-wide association studies. However, for across-population GS, such algorithms are ineffective because they cannot impute markers that are absent from the target set. The second category consists of reference-panel-based methods, such as fastPHASE, IMPUTE, Markov chain haplotyping algorithm (MACH) (Li et al., 2010), and Beagle (Browning et al., 2018). These approaches are usually based on haplotype-clustering algorithms, hidden Markov models (HMMs), and statistical inference to predict missing values in genotype data. Beagle is a widely used imputation tool that applies the Li and Stephens (Li and Stephens, 2003) HMM to generate posterior probabilities for each allele at an imputed marker. MACH is another HMM-based approach that implicitly combines reference and testing samples to infer possible pairs of haplotypes for each individual genotype. Minimac4 is the latest version in the series of genotype imputation software, including MACH and Minimac (Howie et al., 2012; Fuchsberger et al., 2015; Das et al., 2016). However, existing reference-panel-based methods, which were originally developed to meet the requirements of genome-wide association studies (GWASs), cannot directly satisfy the genotype imputation requirements of across-population GS.

An autoencoder is a type of neural network model that has been used to address the problem of missing markers. Previous studies (Chen and Shi, 2019; Song et al., 2022; Kojima et al., 2024) combined autoencoders with convolutional neural networks to construct reference-free imputation models. Dias et al. (2022) developed a pre-trained autoencoder using a large and commonly used reference panel of human chromosome 22. Recently, Mowlaei et al. (2025) reported Split-Transformer with integrated convolutions for genotype imputation (STICI), which achieved high accuracy in regions with highly linked variants. However, previous deep-learning-based genotype imputation methods exhibit several limitations and have not yet been widely adopted as standard practice in the field (Naito and Okada, 2024). A considerable limitation of these deep-learning-based imputation models arises from their reliance on randomly masked training data, which prevents accurate learning of the relationship patterns between markers targeted for imputation and those present in the observed data. In addition, some advanced deep learning methodologies and techniques, including data augmentation, layer normalization, and residual blocks, have not yet been utilized in genotype imputation research. Therefore, deep-learning-based methods remain inferior to conventional HMM-based methods in terms of accuracy, computational efficiency, and universality. Most existing methods focus on reference-free imputation, and related studies are still in an exploratory stage, as indicated by the absence of robust and widely applicable software solutions for general imputation tasks.

GS-Impute was specifically designed to address these limitations. A key breakthrough of GS-Impute is its novel automatic matching algorithm, which successfully resolves the persistent challenge of targeted training with both sporadic and systematic missing data that previous deep-learning-based imputation methods have failed to overcome. Supported by this matching algorithm, GS-Impute automatically detects missing-data patterns and performs customized model training, thus enabling high imputation accuracy for missing genotypes. Additionally, GS-Impute incorporates a data augmentation strategy and several advanced techniques to enhance imputation accuracy, including residual blocks, dynamic learning-rate optimization, and layer normalization. Based on these technical features, GS-Impute facilitates the implementation of low-density across-population GS (LA-GS).

In this study, datasets from two key crops (rice and maize) were used to evaluate the imputation accuracy of GS-Impute and demonstrate the effectiveness of LA-GS in crops. The primary research objectives were to (1) propose the LA-GS strategy, which is suitable for implementing crop GS with low-density markers; (2) introduce GS-Impute, a newly developed imputation method and corresponding software designed to support LA-GS; and (3) conduct initial case studies using rice and maize datasets to evaluate predictability and validate the feasibility of across-population GS under low-density marker conditions.

## Results

### The LA-GS strategy

**Figure 1:**
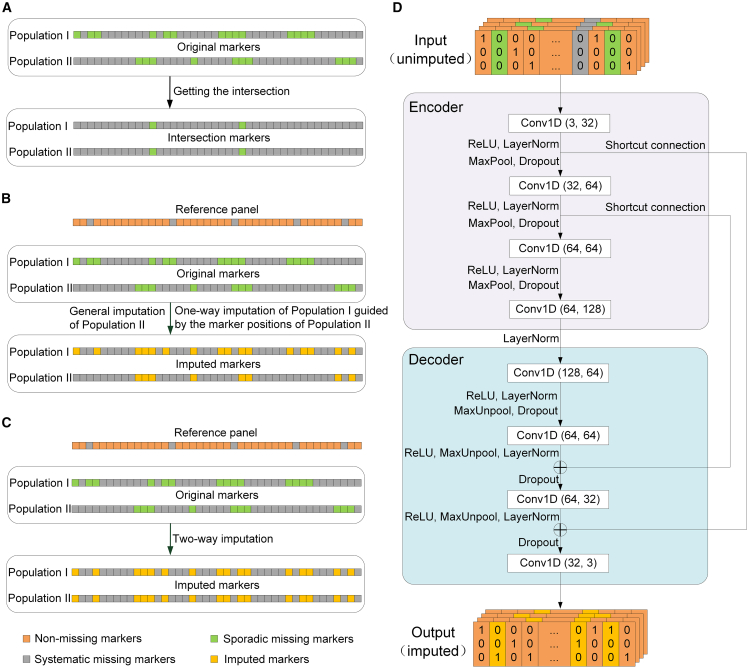
Strategy of low-density marker imputation for across-population GS and the architecture of the neural network model GS-Impute. **(A)** Obtaining the intersection of markers between population I and population II. **(B)** Obtaining the marker set for each population via one-way reconstructive imputation of population I guided by the marker positions of population II, along with general imputation of population II. **(C)** Obtaining the marker set for each population via two-way reconstructive imputation, which consists of two independent one-way imputations. **(D)** Architecture of the neural network model GS-Impute. The model contains eight one-dimensional convolutional layers. The number of input channels in each convolutional layer is 3, 32, 64, 64, 128, 64, 64, and 32, respectively. The first layer receives an *n* × *p* × 3 array, where *n* denotes the number of individuals and *p* indicates the number of markers per individual. Each convolutional layer is configured with a kernel size of 15, a stride of 1, and padding equal to half the kernel size.

Here, we proposed the LA-GS strategy (Figures 1A–1C), which is suitable for implementing crop GS with low-density markers. LA-GS includes one-way imputation and two-way imputation, thereby representing a viable strategy for GS under low-density marker conditions. One-way imputation is a reconstructive process in which the genotype file of one population (population I) is imputed using a reference panel, guided by the position file of another population (population II). Two-way imputation refers to the process in which two separate one-way imputations are performed. In this study, we analyzed three schemes of marker uniformity for across-population GS in crops and propose the LA-GS strategy, which imputes genotyped loci shared across all populations involved in the GS framework. Furthermore, we developed a new neural network model—GS-Impute—and its corresponding software to support the LA-GS strategy and provide breeders with an integrated, one-step solution.

### Comparisons of imputation accuracy with state-of-the-art methods

**Figure 2:**
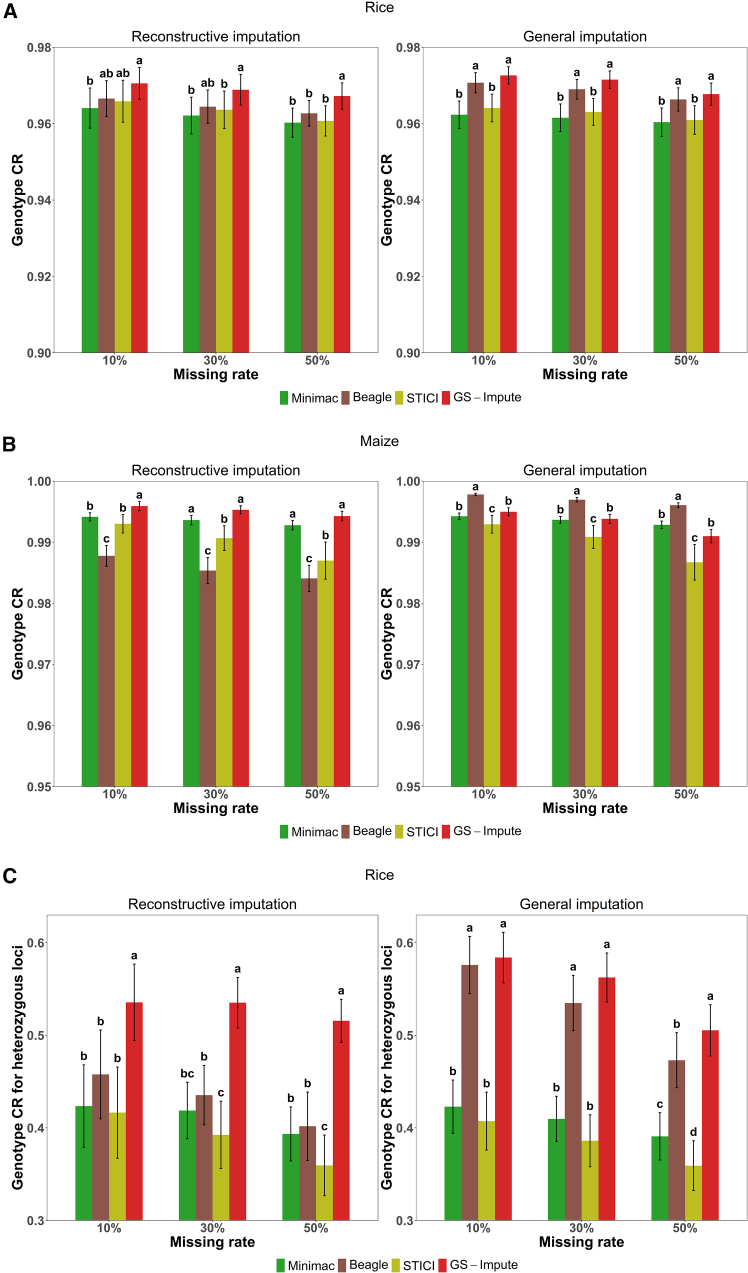
Genotype CR (mean ± SD across all chromosomes) with Tukey’s honestly significant difference (HSD) multiple-comparison test results For rice and maize, results from the 10 and 12 chromosomes were considered independent samples, respectively. The corresponding mean values, SDs, and HSD test results are presented. **(A)** Genotype CR for all loci in the testing set of 120 rice samples. **(B)** Genotype CR for all loci in the testing set of 100 maize samples. **(C)** Genotype CR for heterozygous loci in the testing set of 120 rice samples. Genotype CR represents the proportion of correctly imputed genotypes among all originally missing genotypes.

For the testing sets of 120 rice samples and 100 maize samples, the imputation accuracy of GS-Impute was compared with Minimac4, Beagle5.4, and STICI. The genotype concordance rate (CR) (mean ± SD across all chromosomes) for datasets with missing rates of 10%, 30%, and 50% is shown in Figures 2A and 2B. The genotype *r*^2^ (mean ± SD across all chromosomes) is shown in Figure S1. Regardless of the imputation method utilized, a consistent inverse relationship was observed between missing rate and imputation accuracy (measured by both CR and *r*^2^), such that higher missing rates led to reduced accuracy. For the rice dataset, the mean accuracy of GS-Impute was consistently higher than that of Minimac, Beagle, and STICI across all test scenarios. Concerning the maize dataset, GS-Impute showed optimal performance for reconstructive imputation while exhibiting slightly lower accuracy than Minimac for general imputation. Thus, while maintaining superior accuracy in handling systematic missing data, GS-Impute provides imputation accuracy comparable to that of Minimac and Beagle for sporadic missing data. Notably, GS-Impute achieved higher relative accuracy under systematic missing patterns than under sporadic missing patterns. In the context of systematic missing patterns, where all testing samples share identical missing positions, the masked marker generation algorithm can simulate the true missing pattern of the testing set more accurately than under sporadic missing conditions. Consequently, GS-Impute shows greater imputation accuracy in such systematic scenarios. With respect to genotype imputation approaches, a major challenge in across-population GS is the inherent coexistence of systematic and sporadic missing patterns. These results indicate that GS-Impute can simultaneously address both types of missingness, demonstrating strong robustness under complex missing-data conditions.

### Ablation experiments

**Figure 3:**
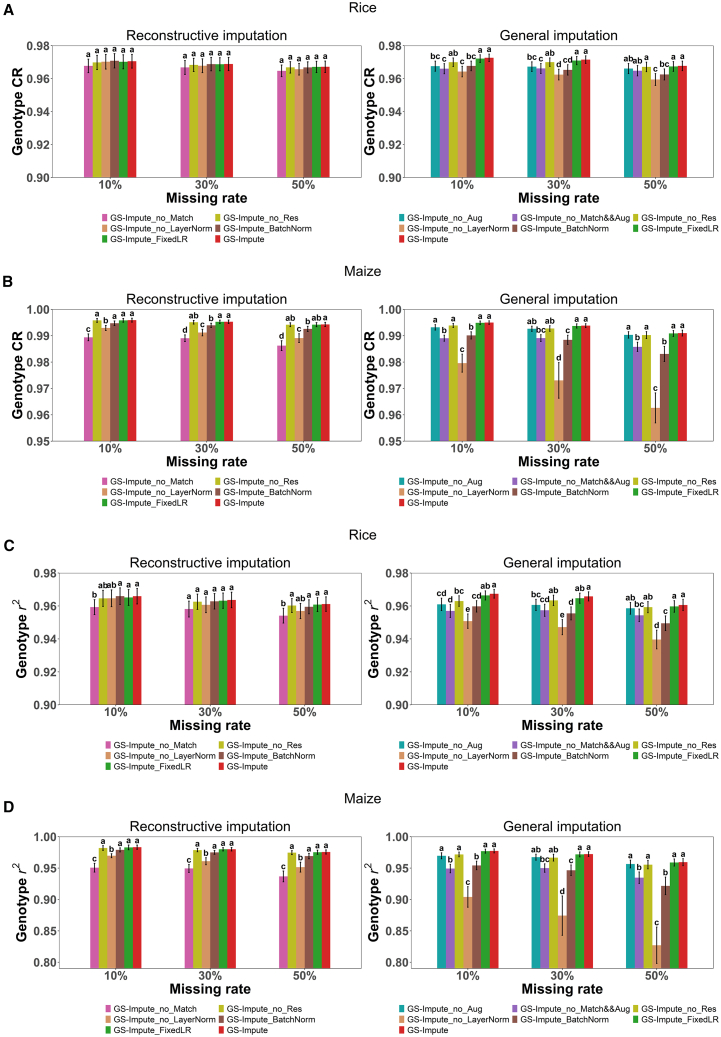
Genotype CR and *r*^2^ (mean ± SD across all chromosomes) with Tukey’s HSD multiple-comparison test results from ablation experiments For rice and maize, results from the 10 and 12 chromosomes were considered independent samples, respectively. The corresponding mean values, SDs, and HSD test results are presented. **(A)** Genotype CR for all loci in the testing set of 120 rice samples. **(B)** Genotype CR for all loci in the testing set of 100 maize samples. **(C)** Genotype *r*^2^ for all loci in the testing set of 120 rice samples. **(D)** Genotype *r*^2^ for all loci in the testing set of 100 maize samples. Genotype CR represents the proportion of correctly imputed genotypes among all originally missing genotypes. Genotype *r*^2^ is calculated as the squared correlation between imputed and true genotypes. GS-Impute_no_Match: version of GS-Impute without automatic matching. GS-Impute_no_Res: version of GS-Impute without residual blocks. GS-Impute_no_LayerNorm: version of GS-Impute without layer normalization. GS-Impute_BatchNorm: version of GS-Impute with batch normalization. GS-Impute_FixedLR: version of GS-Impute without a dynamic learning rate. GS-Impute_no_Aug: version of GS-Impute without data augmentation. GS-Impute_no_Match&&Aug: version of GS-Impute without automatic matching or data augmentation.

To clarify the contributions of multiple techniques incorporated in GS-Impute, ablation experiments (Figure 3) were performed on automatic matching, data augmentation, residual blocks, dynamic learning rate, and layer normalization. In the reconstructive imputation scenario, GS-Impute does not utilize data augmentation techniques; thus, no comparison was conducted between settings with and without augmentation. The version of GS-Impute without automatic matching shows the worst performance among all comparative methods, and the difference is statistically significant in most cases. This result clearly demonstrates that automatic matching is the most critical and indispensable component of GS-Impute. Although the contributions of the other techniques are not always statistically significant, their positive effects remain observable to varying degrees based on the average accuracy metrics. In the general imputation scenario, data augmentation in GS-Impute is implemented on the basis of the matching algorithm; therefore, disabling matching also disables augmentation. The GS-Impute version without layer normalization displays the worst performance, whereas the version that utilizes commonly applied batch normalization shows slightly better results. The version without automatic matching and data augmentation also performs very poorly. These results indicate that automatic matching, data augmentation, and layer normalization are essential to improve accuracy in this scenario.

### Imputation accuracy for specific alleles

**Figure 4:**
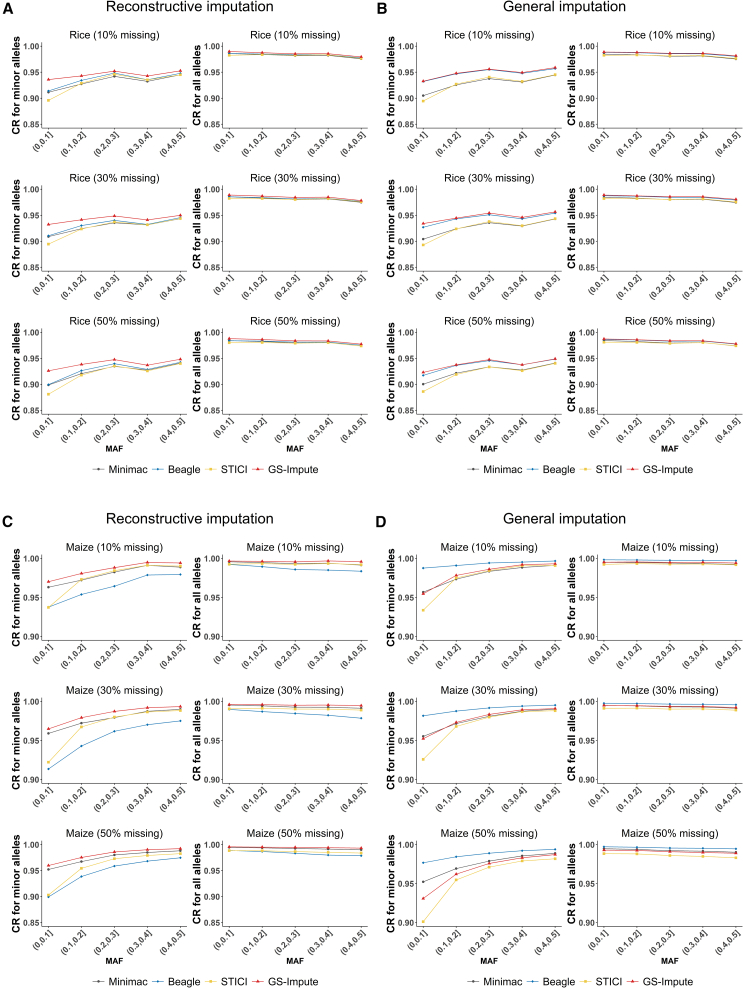
Imputation CR for minor and all alleles across multiple MAF intervals in the whole genome **(A)** Allelic CR for reconstructive imputation in the testing set of 120 rice samples. **(B)** Allelic CR for general imputation in the testing set of 120 rice samples. **(C)** Allelic CR for reconstructive imputation in the testing set of 100 maize samples. **(D)** Allelic CR for general imputation in the testing set of 100 maize samples. Allelic CR represents the proportion of correctly imputed alleles among all originally missing alleles.

To assess the imputation accuracy for alleles with different minor-allele frequencies (MAFs), CRs were evaluated for minor alleles and for all alleles across multiple MAF intervals (Figure 4). In both rice and maize, the CR for minor alleles tended to increase as MAF increased. In contrast, the CR for all alleles tended to decrease with increasing MAF; accuracy at low MAF values was largely determined by the high imputation accuracy of major alleles. Genotype *r*^2^ across multiple MAF intervals was also calculated, and the results (Figure S2) followed a pattern similar to that of the CR for all alleles.

Imputation accuracy was also investigated for heterozygous loci. A locus was considered concordant only when both alleles were imputed correctly. In this study, the samples in the testing sets of both maize and rice were pure lines. The original genotype file of maize treated all markers as homozygous, and heterozygous markers were excluded from the data. In the rice dataset, heterozygous genotypes were distinguished. Therefore, only the CR for heterozygous loci in rice was validated. Additionally, given that the true genotype value is constant across all heterozygous loci, genotype *r*^2^ could not be calculated in this case and was excluded from the comparative analysis. As shown in Figure 2C, GS-Impute achieved the highest CR in all cases. However, the CR for heterozygous loci remained lower than the overall CR dominated by homozygous markers, suggesting that imputation based on homozygous loci is more reliable. Indeed, the heterozygous genotypes of hybrids in this study were derived from the homozygous genotypes of their parents.

### Predictability of across-population GS with different schemes

**Figure 5:**
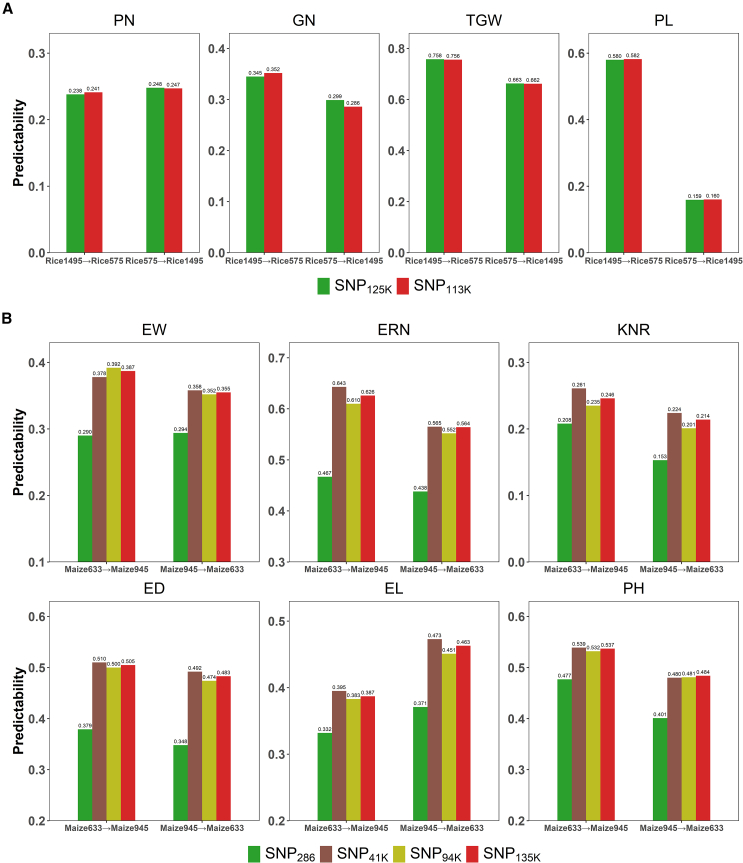
Predictability of across-population GS using GBLUP **(A)** Predictability of across-population GS for rice. The GS scheme Rice1495→Rice575 indicates the use of Rice1495 as the training set to predict Rice575 and vice versa. The marker set SNP_125K_ corresponds to A_r_∩B_r_, and the marker set SNP_113K_ corresponds to B_r_∩P_r_ (where A_r_ denotes the marker set of Rice575, B_r_ denotes the marker set of Rice1495, and P_r_ denotes the marker set of the rice reference panel). SNP_113K_ was generated by first performing one-way reconstructive imputation on the low-density markers of Rice575 using B_r_ as the guide, followed by general imputation on B_r_. **(B)** Predictability of across-population GS for maize. The GS scheme Maize633→Maize945 indicates the use of Maize633 as the training set to predict Maize945, and vice versa. The marker set SNP_286_ corresponds to A_m_∩B_m_, the marker set SNP_41K_ corresponds to B_m_∩P_m_, the marker set SNP_94K_ corresponds to A_m_∩P_m_, and the marker set SNP_135K_ corresponds to (A_m_∪B_m_)∩P_m_ (where A_m_ denotes the marker set of Maize945, B_m_ denotes the marker set of Maize633, and P_m_ denotes the marker set of the maize reference panel). SNP_41K_ was generated by performing one-way reconstructive imputation on A_m_ using B_m_ as the guide, followed by general imputation on B_m_. SNP_94K_ was generated by performing one-way reconstructive imputation on B_m_ using A_m_ as the guide, followed by general imputation on A_m_. SNP_135K_ was generated by performing two-way reconstructive imputation.

To evaluate the efficacy of across-population GS, we compared predictability between two distinct scenarios. One scenario was based on LA-GS, and the other was based on GS without imputation. For rice, using 124 803 SNPs (SNP_125K_) shared between two populations composed of 575 hybrids (Rice575) and 1495 hybrids (Rice1495), GS was performed on four traits; GS using SNP_125K_ served as the reference condition. Assuming that Rice575 contained only low-density markers, 120 000 SNPs were randomly selected from the 12 chromosomes, completely non-overlapping with the 201 756 SNPs in Rice1495. In this scenario, where no shared loci existed between Rice575 and Rice1495, across-population genomic prediction was impossible without imputation of missing markers. In this study, 113 417 SNPs (SNP_113K_) were generated through a one-way reconstructive imputation process, in which the 120 000 SNPs of Rice575 were imputed using the 201 756 SNPs of Rice1495 as a guide. GS was subsequently performed using the imputed dataset. As shown in Figure 5A, after use of genomic best linear unbiased prediction (GBLUP) (VanRaden, 2008), predictabilities in the two scenarios were very similar, indicating that across-population prediction using low-density markers with imputation can achieve results comparable to those obtained with high-density markers, which is impossible without imputation. For maize, GS was initially conducted on six traits using 286 shared SNPs (SNP_286_) across two populations: 945 hybrids (Maize945) with 108 541 SNPs and 633 hybrids (Maize633) with 43 661 SNPs. We then constructed three SNP sets: SNP_41K_ (41 170 SNPs) through one-way imputation of Maize945 guided by the 43 661 SNPs of Maize633; SNP_94K_ (93 752 SNPs) through one-way imputation of Maize633 guided by the 108 541 SNPs of Maize945; and SNP_135K_ (134 654 SNPs) through two-way imputation. GS was performed separately using SNP_41K_, SNP_94K_, and SNP_135K_. The GBLUP results in Figure 4B show that across-population predictability with SNP_286_ was very low. Thus, GS cannot be effectively performed when very few intersection markers are shared between two low-density populations. Notably, for ear weight, ear row number, kernel number per row, ear diameter, ear length, and plant height, the predictabilities obtained with SNP_41K_, SNP_94K_, and SNP_135K_ considerably exceeded those with SNP_286_ by averages of 26.8%, 31.1%, 27.5%, 35.9%, 21.0%, and 15.9%, respectively. These results indicate that GS-Impute can strongly enhance across-population GS with low-density markers. However, SNP_135K_ showed no clear advantage over SNP_94K_ or SNP_41K_, and its predictability was generally intermediate between these two marker sets, indicating that two-way imputation can enhance robustness compared with one-way imputation by incorporating a larger number of markers. In principle, two-way imputation serves as a balancing mechanism that integrates genetic information from two independent marker sets. This approach combines linkage disequilibrium patterns from different populations. Although this strategy may sacrifice the highest accuracy achievable within a single population, it improves model robustness across diverse genetic backgrounds by reducing reliance on population-specific linkage patterns. This characteristic is suspected to explain the intermediate performance of SNP_135K_ while demonstrating the method’s value in improving generalizability.

Across-population prediction was performed using not only GBLUP but also least absolute shrinkage and selection operator (LASSO) (Friedman et al., 2010) and BayesB (Meuwissen et al., 2001) with rice datasets (SNP_125K_ and SNP_113K_) and maize datasets (SNP_286_ and SNP_135K_). The results (Tables S1 and S2) demonstrated that the three methods achieved comparable accuracy, whereas GBLUP showed greater robustness. These findings also indicated that the observed improvement in performance primarily arose from imputation rather than dependence on a specific GS method. Additionally, the predictability of within-population GS for rice (using SNP_125K_) and for maize (using SNP_135K_), as determined by GBLUP, is presented in Tables S3 and S4. In all cases, the predictability of across-population GS was lower than that of within-population GS. Given that the same set of markers was used, this reduction is evidently attributable to differences in population structure and environmental conditions. Traits showing higher predictability in within-population GS generally tend to exhibit higher predictability in across-population GS; importantly, this relationship is not absolute. Contributing factors likely include the complexity of environmental variation and the lack of relevant environmental data for prediction. For example, both Rice575 and Rice1495 display high predictability of within-population GS for the panicle length trait. However, in across-population GS, Rice1495→Rice575 (using Rice1495 as the training set to predict Rice575) achieves satisfactory predictability, whereas Rice575→Rice1495 (the reciprocal approach) performs poorly.

### Computational efficiency

Using a 24-core AMD CPU (EPYC 7352 2.3 GHz), 250 GB RAM, and an NVIDIA GTX 4090 GPU, the computation times for GS-Impute, Beagle, Minimac, and STICI were recorded. For the reconstructive imputation of 10 chromosomes in maize, the times required for Beagle, Minimac, STICI, and GS-Impute were approximately 3, 1, 721, and 61 min, respectively. For the reconstructive imputation of 12 chromosomes in rice, the times required for Beagle, Minimac, STICI, and GS-Impute were approximately 6, 2, 1225, and 418 min, respectively. These results show that the computation time was considerably longer for GS-Impute than for Beagle or Minimac but substantially shorter than that for STICI. We note that the recorded time does not include the generation of a reference panel subset, whose loci represent the intersection between the union loci of two populations and the loci of the original reference panel. In practical reconstructive imputation with low-density markers, breeders encounter considerable difficulty when generating reference panel subsets because the process typically requires extraction of chromosomal and marker information from multiple large genotype files with heterogeneous formats, followed by complex merging operations to obtain the necessary subset data. This process is relatively complex; in many cases, the memory capacity and software configuration of conventional personal computers cannot satisfy computational requirements. The GS-Impute software developed in this study can accomplish the procedure in a single step. In particular, for the CSV-formatted chip data widely utilized by breeders, GS-Impute provides effective imputation capacity, whereas most conventional methods are restricted to VCF-format inputs. Furthermore, GS-Impute can automatically segment large genotype files (including reference panels), which enables imputation of massive datasets on personal computers. Although the marker imputation step in the proposed approach requires extended computation time, the overall workflow demonstrates superior practical efficiency.

## Discussion

Accuracy is clearly an important criterion for evaluating the performance of various imputation methods. The statistics *r*^2^ and CR have been widely used in previous studies of imputation methods (Li et al., 2010; Fuchsberger et al., 2015; Das et al., 2016; Browning et al., 2018; Dias et al., 2022). We note that genotype codes are discrete values; the primary purpose of imputation in the present study was to facilitate subsequent GS. Conventional GS methods typically predict potential phenotypes according to estimated genetic effects of alleles at each locus (Wang et al., 2015). CR provides an intuitive metric, representing the proportion of correctly imputed genotypes that are incorporated into GS models for training or prediction. Therefore, CR was used as the main measure of accuracy in this study. Additionally, the accuracy of GS-Impute was compared with the latest versions of three benchmark tools: Minimac4, Beagle5.4, and STICI. In a recent study, DPImpute (Zheng et al., 2025), a two-step imputation pipeline incorporating Beagle5.1, achieved accurate SNP genotyping under ultra-low coverage whole-genome sequencing, small testing sample sizes, and limited reference populations. Notably, in our preliminary experiments, Beagle5.4 demonstrated considerably higher accuracy than Beagle5.1 for certain datasets. Although GS-Impute showed only modest absolute improvements in accuracy relative to Minimac4, Beagle5.4, and STICI (Figures 2A and 2B), these results nevertheless confirm the superiority of the proposed algorithm, particularly given the high baseline accuracy.

To meet the needs of GWASs, imputation accuracy for minor alleles has historically received substantial attention. However, many existing genotype imputation methods show reduced accuracy when inferring genotypes of genetic variants with low frequency (Clouard and Nettelblad, 2024). Previous studies (Fuchsberger et al., 2015; Rubinacci et al., 2020, 2021; Stahl et al., 2021; Dias et al., 2022) indicated a large decline in accuracy for rare variants with low MAF, and the performance of GS-Impute for rice and maize followed a similar pattern. Nevertheless, imputation was used to support GS in the present study. Conventional GS studies often exclude markers with very low MAF (Yin et al., 2020; Liang et al., 2023; Wang et al., 2023); in contrast, the classical method GBLUP utilized in this study assumes equal variance across all loci and assigns equal importance to each locus. This approach is more robust than many alternative methods because it does not depend on estimated marker effects (Xu et al., 2014). Consequently, it imposes less stringent requirements on the accuracy of rare-allele imputation compared with GWAS analyses. Given these factors, the present study extended the evaluation beyond minor alleles to comprehensively assess imputation accuracy across all alleles with various MAF values. The imputation performance for rare variants demonstrated acceptable accuracy, with a positive correlation between increasing accuracy and increasing MAF; this result aligned with findings from previous studies (Pei et al., 2008; Song et al., 2022; Zhang et al., 2023).

The genotype imputation performed in this study represents an early step in across-population GS. Because of correlations among nearby markers, many untyped SNPs within a genomic region are highly correlated with adjacent typed SNPs (Servin and Stephens, 2007). Most genotype imputation methods rely on linkage information between markers, but the selection of appropriate data is equally important to ensure imputation accuracy. For breeders, one major objective of GS is the prediction of new-generation hybrid performance using historical data (Lorenzi et al., 2024). However, in hybrid varieties, the genotypes at each locus are artificially controlled, which makes direct imputation unsuitable. A more effective strategy is to impute parental line genotypes, then infer hybrid genotypes. For rice, the two populations consisted of 575 hybrids (derived from crosses among 120 inbred lines) and 1495 hybrids. We obtained SNP data for the 1495 hybrids, but those of their parents were unavailable. Therefore, one-way reconstructive imputation was necessary; 120 000 low-density markers distributed across the whole genome of 120 inbred samples were used as the testing set to simulate the requirement for imputation in across-population GS. For maize, the two populations consisted of 945 hybrids (derived from crosses among 266 inbred lines) and 633 hybrids (derived from crosses among 342 inbred lines). Because parental genotypes were available, two-way reconstructive imputation could be performed. The genotype data of the 266 inbred lines were stored in HMP format (which can be converted to VCF format), whereas the genotype data of the 342 inbred lines were stored in CSV format. Although GS-Impute can process CSV genotype files, Minimac, Beagle, and STICI support only VCF format. To enable a fair comparison of accuracy among methods, 93 752 markers from 100 samples with a low missing rate among the 266 samples were selected as the testing set to approximate the requirements of across-population GS.

Most neural-network-based imputation methods proposed in previous studies (Chen and Shi, 2019; Song et al., 2022) were reference-free, whereas GS-Impute—developed in the present study—is a reference-based method. Although the proposed framework can be modified to operate in a reference-free mode, the present design focuses on reference-based imputation to support across-population GS. Therefore, GS-Impute was compared only with three widely used reference-based methods (Minimac, Beagle, and STICI), rather than with any reference-free models. Marker position uniformity is a fundamental requirement for across-population GS in crops. The most straightforward approach involves utilizing shared marker positions from identical gene chips or aligned markers derived from dense genotypes, as illustrated in Figure 1A. However, existing genotype datasets typically are not generated for across-population GS, and their marker density may be insufficient to meet these requirements. Under the more general condition of low-density markers, genotype data of crop varieties can be imputed to dense marker sets or even to sequence level using a reference population. In practice, the selection of suitable schemes and tools remains difficult. Reconstructive imputation with GS-Impute, based on the LA-GS strategy, provides an efficient and cost-effective solution. The one-way imputation and two-way imputation schemes proposed in this study correspond to two typical scenarios in across-population GS for crops. The former applies to cases in which genotype data are available for only one inbred population, whereas the latter requires genotype data from inbred lines of both populations. In either scenario, GS-Impute provides comprehensive support for the key imputation processes required in GS.

Most crop breeders tend to focus on genetic research and field management; they are unfamiliar with the processing of large-scale biological data. Existing imputation methods are often tailored to the requirements of GWAS, and it is difficult to perform the reconstructive imputation and related data cleaning necessary for across-population GS. The GS-Impute software proposed in the present study was designed to address this problem. Using this tool, VCF-, HMP-, or CSV-format genotype files generated by breeders can be accurately imputed on a personal computer; integer-encoded genotype files can be output directly, which facilitates subsequent across-population GS analyses.

Although GS methods can effectively predict crop phenotypes, most previous studies have focused on specific, isolated populations. Differences in genetic materials, laboratory protocols, and sequencing platforms frequently produce substantial discrepancies in marker positions, which prevent the integration of these markers into unified prediction models and ultimately hinder improvements in GS efficiency. Over the past 15 years, reference panels have become larger and more diverse (Cavinato et al., 2024). Recently, genotype imputation in crops has greatly benefited from the increasing availability of publicly accessible genetic resources (Gao et al., 2021). Across-population GS based on genotype imputation can improve data utilization efficiency while facilitating the construction of more representative large-scale training populations derived from various environments. This approach allows breeders to reconstruct missing genotypes of certain populations at specific chip loci, complete the consistent conversion of marker positions, enable the sharing and use of across-population GS information, and promote broader application of GS in crops.

Moreover, the predictability of GS increases almost linearly with training set size (VanRaden et al., 2009). In a previous study (Wang et al., 2015), when the number of individuals in the training set increased from 300 to 539, the average coefficient of determination of five GS models increased by 26%. Larger sample sizes that include both phenotypic and genotypic data provide more comprehensive information, which facilitates more accurate estimation of genetic effects at each marker locus and consequently improves GS predictability (Goddard, 2009). Although the present study primarily examined mutual prediction between two populations based on genotype imputation, the proposed strategy can be readily extended to multiple populations. Beyond pairwise prediction, this approach enables the construction of comprehensive training populations that incorporate diverse genetic resources, thereby enhancing the efficacy of GS in crop breeding programs.

## Methods

### Genotypes for imputation evaluation

In this study, two datasets, rice and maize, were used to evaluate the accuracy of the imputation methods. For rice, 4 897 277 genome-wide SNPs from 3240 samples in the Plant-ImputeDB platform (Gao et al., 2021) were used as the reference panel. Across the whole genome of 120 inbred lines (Wang et al., 2017) from Wuhan University, 3 017 017 loci were genotyped via next-generation sequencing. In total, 120 000 non-missing markers intersecting with the reference panel were selected as the testing set, in which 10 000 markers were randomly selected from each chromosome. For maize, 35 073 758 genome-wide SNPs from 1210 samples in the Plant-ImputeDB platform were used as the reference panel. For the imputation test, 108 541 SNPs across the entire maize genome of 266 elite inbred lines (Zhang et al., 2025) (Maize266), obtained via genotyping-by-sequencing technology, were used. The original marker missing rate of this dataset was 10.5%. To reduce interference in the evaluation of imputation accuracy, 93 752 markers intersecting with the reference panel were selected from 100 samples with a low missing rate (5.0%) as the testing set, and the original missing markers were excluded when calculating imputation accuracy. For the testing sets of rice and maize, 10%, 30%, and 50% of markers were masked using two distinct patterns to generate missing data for imputation: a systematic missing pattern, in which certain markers were absent across all samples, and a sporadic missing pattern, in which missing markers were randomly distributed within each sample. In this study, we defined the imputation process under the systematic missing pattern as reconstructive imputation, whereas imputation under the sporadic missing pattern was considered general imputation. We extracted genotype data at the corresponding loci for all samples from the reference panel according to marker positions in the testing set for model training, then conducted imputation in the testing set. Imputation accuracy was quantified using CR and genotype *r*^2^. Genotype CR was defined as the proportion of correctly imputed genotypes among all originally missing genotypes. A genotype was considered concordant only when both alleles at a locus were imputed correctly. Allelic CR was defined similarly for imputed versus true alleles. Genotype *r*^2^ was calculated as the squared correlation between imputed and true genotypes.

### Plant materials for across-population GS

Across-population GS was applied to rice and maize in the present study. For rice, two datasets were used. One dataset comprised 575 hybrids derived from crosses among the 120 inbred lines described above. The inbred parents were genotyped at 3 017 017 loci via next-generation sequencing. The phenotypes of the 575 hybrids were evaluated in two environments in China, with two replicates in each environment. The second dataset comprised 1495 hybrids (Huang et al., 2015) from the Shanghai Institutes for Biological Sciences; genotype data of 201 756 SNPs were available. Four traits common to both populations were used for genomic prediction: productive panicle number per plant, grain number per panicle, thousand-grain weight, and panicle length.

Similar to the rice analysis, maize evaluation was conducted using two distinct datasets. One dataset was composed of partial diallel crosses among the lines in Maize266, which were performed during the 2018 maize growing season in Yangzhou and Taian, China, with two replicates in each environment. Genotypes of the hybrids were inferred from the 108 541 SNPs of their parental inbred lines. The second dataset was composed of 633 hybrids derived from crosses among 342 maize inbred lines (Maize342), which were evaluated during the 2021 and 2022 growing seasons in Zhenjiang, China, with two replicates per environment. The 40K maize liquid array was used to genotype the 342 inbred lines at 43 661 loci. Regardless of reciprocals, six traits of Maize945 and Maize633 were used in this study for across-population GS: ear weight, ear row number, kernel number per row, ear diameter, ear length, and plant height.

### Strategy of low-density marker imputation for across-population GS

In the present study, the case of two crop populations served as an example to investigate across-population GS with low-density markers. For two crop populations consisting of pure lines, the set of markers in population I was denoted as A and the set of markers in population II was regarded as B. If imputation is not performed, across-population GS can use only the intersection of A and B (A∩B) (Figure 1A).

In most scenarios, the intersection of low-density marker sets is insufficient to meet genome-wide marker requirements for effective GS, making this approach impractical. The application of imputation technology substantially alters this situation. By leveraging established imputation methods such as Minimac and Beagle, marker sets can be generated for each crop population that correspond to the marker set of the reference panel. Although this strategy seems reasonable, our results indicated that imputation accuracy shows an inverse relationship with marker missing rate. Consequently, imputation to high-density marker sets or sequence-level resolution may reduce the reliability of across-population GS. Additionally, panel-scale marker datasets are often difficult to manage on personal computers with limited memory, and many GS models encounter challenges such as decreased efficiency and overfitting when processing large-scale marker datasets, leading to substantial obstacles for breeders attempting to implement across-population GS.

To address the issues described above, this study introduces a low-density marker imputation strategy termed LA-GS, which imputes only the genotyped markers shared among all populations involved in across-population GS. The software GS-Impute 1.0 was developed to support this strategy. In crop GS breeding, this strategy is applicable to two typical scenarios. The first scenario is one-way reconstructive imputation. This is a reconstructive process in which the genotype file of one population (population I) is imputed using a reference panel, guided by the marker position file of another population (population II). The output file contains all samples from population I and includes the marker set defined as (A∪B)∩P, where P represents the marker set in the reference panel. For instance, the two rice populations analyzed in the present study include both pure lines and hybrids, but only the pure-line population (Rice120) is suitable for imputation guided by the marker positions of the hybrid population (Rice1495). After one-way reconstructive imputation of population I and general imputation of population II (which retains only the intersecting markers between population II and the reference panel), the marker set shared by populations I and II is B∩P (Figure 1B). The second scenario, two-way reconstructive imputation, refers to the performance of two independent one-way imputations. First, the genotype of population I is imputed according to the marker positions of population II, and then the genotype of population II is imputed according to the marker positions of population I. This bidirectional strategy allows both populations to be genotyped on a shared and expanded marker set. For example, both maize parental populations in this study (Maize266 and Maize342) comprise pure lines; the genotype of each population can be imputed according to the marker positions of the other population using a high-density reference panel. In this process, one-way imputation is performed in both directions, producing the marker set B∩P guided by the positions of B and the marker set A∩P guided by the positions of A. After completion of both one-way imputations, the marker set (A∪B)∩P (Figure 1C), derived from two-way reconstructive imputation, can be obtained.

### Genotype encoding

Let A_1_ and A_2_ denote the two alleles at a SNP locus, where A_1_ represents the allele identical to the reference genome at that locus. For preprocessing of genotype data in this study, we implemented a numerical coding system, in which values of 1, 2, and 3 represent the genotypes A_1_A_1_, A_1_A_2_, and A_2_A_2_, respectively; missing markers are encoded as 0. To make the distance calculation between genotype features more appropriate, we utilized one-hot encoding to transform numerical values into binary vectors before entering training data into the neural network model. The one-hot encoding transformation follows these rules:

$$\left\{ \begin{array}{c} \left[\;0,0,0\right]\;\text{missing}\;\text{markers}\\ \left[\;1,0,0\right]\;A_{1}A_{1}\\ \left[\;0,1,0\right]\;A_{1}A_{2}\\ \left[\;0,0,1\right]\;A_{2}A_{2} \end{array}\right.\text{.}$$

### Neural network model

GS-Impute is a neural network framework based on a residual convolutional denoising autoencoder, and its architecture comprises eight one-dimensional convolutional layers (Figure 1D). The number of input channels in each convolutional layer is 3, 32, 64, 64, 128, 64, 64, and 32, respectively. The first layer receives an *n* × *p* × 3 array, where *n* represents the number of individuals and *p* represents the number of markers per individual. Each convolutional layer is configured with a kernel size of 15, a stride of 1, and padding equal to half the kernel size. In the model architecture, both maxpooling and upsampling are applied. Maxpooling utilizes downsampling to reduce dimensionality by applying a max filter to non-overlapping subregions of the previous layer. Upsampling is the reverse operation of maxpooling; it increases dimensionality by repeating data along the corresponding axis. The model was trained using the cross-entropy loss function, with a batch size of 64. Optimization was performed using the Adam optimizer with an initial learning rate of 1e−03, epsilon of 1e−08, and weight decay of 1e−05.

To enhance imputation accuracy, a sliding-window approach was implemented, in which markers on each chromosome were trained in segments during genotype imputation. In our experiments, a smaller window size (several hundred markers) slightly improved imputation accuracy but increased computation time model due to the larger number of training iterations. To balance accuracy and efficiency, the window size was set to 1000 in this study. When approaching the end of a chromosome, the training window began from the last marker and extended backward to include 1000 markers. For the terminal region of the chromosome, training and imputation results obtained from this reverse window were used to impute the remaining markers. This design ensured that terminal regions with fewer than 1000 markers were processed in a manner consistent with the other windows.

Compared with existing deep-learning-based marker imputation models, GS-Impute incorporates many important technologies that have rarely been applied in this field, including residual blocks (He et al., 2016), dynamic learning-rate optimization, layer normalization, an automatic matching algorithm, and data augmentation, with the goal of improving model speed and accuracy.

In marker imputation, we used a network with residual blocks, characterized by the addition of an identity mapping x. For a stacked structure, when the input is x, the underlying mapping to be fityrf is denoted as H(x). The residual network allows the stacked layers to fit a residual mapping defined as F(x) = H(x) – x; the original mapping becomes F(x) + x. Although both forms are theoretically able to be asymptotically approximate, the residual mapping is easier to optimize than the original mapping, thus addressing degradation problems caused by increased network depth. The residual block facilitates optimization by allowing faster convergence during early training. In our experiments, the model typically converged after slightly more than 100 epochs. Therefore, the maximum number of epochs was set to 150, which is considerably lower than values commonly used in most deep neural networks. Additionally, our model was designed to save the learned parameters in an internal state dictionary when an improvement was detected (according to the monitored loss metric) during training, ensuring that the latest optimal model was retained. After completion of training, the optimal model was loaded to perform prediction on the testing set.

Learning-rate scheduling is a widely adopted technique for enhancing optimization efficiency and model performance. To overcome the limitations of a fixed learning rate in conventional neural networks, the present study implemented a decaying learning-rate strategy, rather than maintaining a constant value. After 100 training epochs, a ReduceLROnPlateau scheduler was utilized to dynamically adjust the learning rate, with a reduction factor of 0.9 and a patience of 0 epochs, while monitoring the loss in minimization mode. Through this strategy, when the network approaches the minimum of the error curve, a smaller learning rate allows the model to learn a better (or even globally optimal) set of weights, although at the cost of reduced learning speed.

GS-Impute uses the ReLU activation function to introduce non-linear transformations in each encoding and decoding layer. Layer normalization (LayerNorm) is used to normalize the distributions of intermediate layers, enabling smoother gradients, faster training, and better generalization accuracy (Xu et al., 2019). In the network architecture, LayerNorm was inserted after ReLU or before each addition of the identity mapping and residual mapping.

The typically limited sample sizes in crop genotype imputation datasets create a substantial risk of overfitting in deep neural network models, which could substantially reduce imputation accuracy when generalizing to new data. Dropout is a regularization technique commonly used to reduce overfitting, whereby a specified proportion of neuron outputs is set to zero during training. In the present study, the dropout rate was determined according to the following empirical formula: dropout rate = 0.3 − (*mr* - 0.1)/8, where *mr* represents the missing rate of genotypes.

More importantly, this study introduced a novel data-adaptive masked marker generation algorithm that incorporates automatic matching between training and testing samples to address a key limitation of conventional deep-learning-based imputation models. After training data had been obtained, we generated missing values within the dataset using the automatic matching algorithm for model learning. Subsequently, imputation was performed on the testing set. This approach specifically resolves the substantial loss of accuracy that occurs when individuals with different missing-marker patterns are imputed.

### Masked marker generation algorithm based on automatic matching of samples

#### For reconstructive imputation

To enable neural network training, a corrupted training dataset containing missing values must be generated and used as input. When multiple testing samples share identical missing-marker patterns, the generation of training samples with corresponding missing-marker distributions is relatively simple. However, in practical imputation tasks, testing samples frequently exhibit both systematic and sporadic missing-marker patterns, which leads to heterogeneous missing positions among samples. In such cases, sequential imputation of testing samples can preserve accuracy, but this compromise greatly increases computational cost and thus is impractical for most applications. To address this issue, before each training step of the GS-Impute model, a corrupted training dataset containing masked marker values was generated using a two-step process. First, the data-adaptive algorithm determined which markers should be masked through automatic matching between training and testing samples. Second, the selected markers were masked (set as missing) in the original training data to create the corrupted dataset used for model training. The detailed procedure is as follows.

1. Numerically encode the genotype matrix of *n* samples in the training set (reference panel) to obtain ***Tr*** and encode the genotype matrix of *m* samples in the testing set to obtain *Te*.
2. Replace the values of missing markers in the testing samples with the mean values of the corresponding markers in the training samples, and obtain the processed testing set $\bar{Te}$.
3. Calculate the Manhattan distance (defined as the sum of absolute differences of numerically encoded values) between training samples and testing samples using ***Tr*** and ***Te***, and obtain the Manhattan distance matrix ***D***_***n***×***m***_.
4. Calculate the average Manhattan distance between each testing sample and all training samples using ***D***_***n***×***m***_.
5. Sort all testing samples in descending order according to the average Manhattan distance calculated in the previous step.
6. For each testing sample arranged in the previous step, select a training sample with the smallest Manhattan distance from the remaining training samples without replacement, then generate missing markers in the training sample at the same positions as in the corresponding testing sample.
7. Repeat the previous steps until all training samples have been selected. At this stage, the generation of masked markers is completed, and the corrupted training set $\tilde{Tr}$ is obtained.

#### For general imputation

During general imputation tasks, sporadic missing marker genotypes are always present in multiple testing samples, and their performance is not determined solely by the imputation accuracy of systematic missing markers, as in reconstructive imputation. Therefore, a more refined training-set construction and masked-marker generation algorithm was designed for general imputation in the present study. Data augmentation refers to methods that construct iterative optimization or sampling procedures through the introduction of unobserved data or latent variables (van Dyk and Meng, 2001). To make full use of non-missing information in the testing data, a data-augmentation scheme was implemented by stacking the original training and testing datasets to improve the training effect. The detailed procedure is as follows.

1. Perform steps 1–7 in the masked marker generation algorithm for reconstructive imputation (described above) to obtain the corrupted training set $\tilde{Tr}$.
2. Impute the missing markers in the testing set ***Te*** using the classical and efficient KNN method, then obtain the KNN testing set ***Te***_*knn*_.
3. Calculate the Manhattan distance between testing samples using ***Te***_*knn*_, and obtain the Manhattan distance matrix ***D***_***m***×***m***_. The diagonal elements of the matrix are then set to twice the maximum value in the matrix to prevent a testing sample from being matched with itself.
4. Calculate the average Manhattan distance for each testing sample using ***D***_***m***×***m***_.
5. According to the average Manhattan distance calculated in the previous step, arrange all testing samples in both descending and ascending order.
6. For each testing sample in the descending and ascending lists, select another testing sample with the smallest Manhattan distance (without replacement) for matching. Next, generate masked markers in the corresponding KNN testing sample at the same positions as those in the arranged testing sample to obtain ${\tilde{Te}}_{knn}$. During masked-marker generation, the original missing positions of the arranged testing sample are excluded from masking in the matched sample.
7. Stack ***Te***_*knn*_ (*m* samples) with the original training set ***Tr*** (*n* samples) to obtain the augmented training set ***Tr***′(*n* + *m* samples). The corrupted sets $\tilde{Tr}$ and ${\tilde{Te}}_{knn}$ are also stacked to produce the augmented corrupted training set $\tilde{{Tr}^{\prime }}$. The augmented sets ***Tr***′ and $\tilde{{Tr}^{\prime }}$ are then used as key inputs to the model.

The pseudocode of the masked marker generation algorithm for both reconstructive and general imputation is provided in the supplemental information. Although the masked marker generation algorithms for reconstructive and general imputation are designed for different scenarios, the two procedures are mutually compatible. This compatibility arises because both algorithms are built on the same matching strategy and neural network model. The main difference is that general imputation utilizes information from known markers in the testing set for data augmentation; in reconstructive imputation, accuracy remains largely unchanged before and after data augmentation. Thus, to reduce computation time, data augmentation is omitted during reconstructive imputation. In practical applications, the general imputation module of the software is intended for standard imputation tasks, where the data to be imputed may contain both sporadic and systematic missing data values. In contrast, the reconstructive imputation module is designed for reconstructive tasks and requires a position file as input. Its imputation procedure can also manage sporadic and systematic missing data simultaneously.

### Development of the GS-Impute software

**Figure 6:**
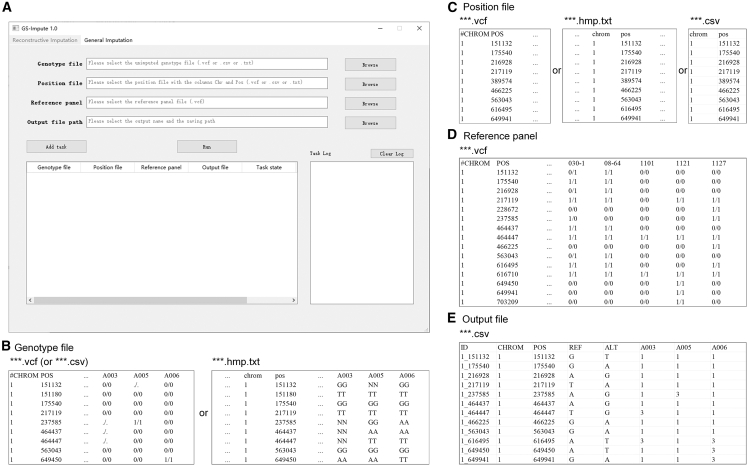
Software interface in the Windows system and input/output format examples of GS-Impute 1.0 for reconstructive imputation **(A)** User interface of GS-Impute 1.0 for reconstructive imputation in the Windows system. **(B)** Example of the genotype file format used as input for reconstructive imputation, where “...” represents omitted nonessential information. The file may contain markers from multiple chromosomes. For genotype files in CSV or TXT format, the column header immediately preceding valid genotype data must be ref, alt, or QCcode. **(C)** Example of the position file format used as input for reconstructive imputation. The file may contain position information from multiple chromosomes. A genotype file containing chromosome and position information can also be used as the position file. **(D)** Example of the reference panel format used as input for reconstructive imputation. The panel must be a VCF file corresponding to a single chromosome, and only markers on that chromosome can be imputed. Missing markers are not permitted in the reference panel. **(E)** Example of the output file format for reconstructive imputation. Genotype values of 1 and 3 represent homozygous variants with two reference alleles and two alternate alleles, respectively. The genotype value of 2 represents a heterozygous variant containing one reference allele and one alternate allele.

GS-Impute 1.0 is an imputation software package designed for GS based on a residual convolutional denoising autoencoder. This program supports both Windows and Linux operating systems; it can efficiently process genotype data in multiple file formats, including VCF, CSV, and TXT. Although GS-Impute does not perform phasing, it is compatible with both phased and unphased genotype data. The software includes two primary functional modules: General imputation and Reconstructive imputation. The General imputation module performs genotype imputation using a reference panel, in accordance with the conventional imputation framework. This function imputes only markers shared by the target dataset and the reference panel; it excludes markers unique to the reference panel. In contrast, the Reconstructive imputation module performs one-way reconstruction, in which the genotype file of population I is imputed using a reference panel, guided by the marker position file of population II. The output file contains all samples from population I and includes the marker set defined as (A∪B)∩P. When two-way reconstructive imputation is required, the same function can be used to impute the genotype file of population II via the reference panel, guided by the marker position file of population I. For reconstructive imputation, the software interface in the Windows system and examples of the input/output formats of GS-Impute 1.0 are shown in Figure 6. Detailed instructions regarding the use of GS-Impute 1.0 are provided in its user manual.

### Genomic prediction model

This study evaluated the accuracy of across-population prediction through three widely used GS methods: GBLUP (VanRaden, 2008), LASSO (Friedman et al., 2010), and BayesB (Meuwissen et al., 2001). Considering the characteristics of these methods and the prediction results obtained, we primarily selected the GBLUP method for presentation. GBLUP is an efficient method that uses whole-genome markers to predict genetic values and phenotypes of interest (VanRaden, 2008). It exploits genomic relationships between training and testing sets to estimate genomic values for unknown individuals. This method is recommended due to its strong robustness when managing the unavoidable errors introduced during imputation of missing markers across populations. Unlike methods that are highly sensitive to individual marker effects, GBLUP relies on a genomic relationship matrix that integrates information from all markers. This strategy effectively averages or reduces noise caused by imperfect genotype imputation, providing more stable and reliable predictions in across-population GS. The model can be written as

*y* = *Xβ* + *Zα* + *ε*,

where *y* is an *s* × 1 vector of hybrid observations, *β* is a vector of non-genetic fixed effects, *X* is an incidence matrix corresponding to the fixed effects *β*, *α* is a vector of random additive regression coefficients for all markers, and *Z* is an *s* × *k* matrix for *k* SNP markers of hybrids that specifies genetic values (−1, 0, and 1 for genotypes A_1_A_1_, A_1_A_2_, and A_2_A_2_, respectively) at each locus. *ε* is a vector of residuals. The breeding value *a* (equal to *Zα*) follows a multivariate normal distribution. It is assumed that $a\;∼\;N\left(0,G_{a}\sigma_{a}^{2}\right)$ and $\varepsilon \;∼\;N\left(0,I_{m}\sigma_{\varepsilon }^{2}\right)$, where $\sigma_{a}^{2}$ is the additive genetic variance, $\sigma_{\varepsilon }^{2}$ is the residual variance, and *G*_*a*_ is the additive genetic relationship matrix. GS models were implemented using the R package predhy (Wang et al., 2025). Predictability was assessed using the Pearson correlation coefficient between the true and predicted phenotypes.

## Data and code availability

Data supporting the findings of this study are available in figshare (https://doi.org/10.6084/m9.figshare.29648240.v1). The source code of GS-Impute is hosted on GitHub (https://github.com/seuwangxin/GS-Impute).

## Funding

This work was supported by grants from the 10.13039/501100012166National Key Research and Development Program of China (2022YFD1201804), the 10.13039/501100001809National Natural Science Foundation of China (32561143291, 32061143030, and 32170636), the Seed Industry Revitalization Project of Jiangsu Province (JBGS[2021]009), and the Key Research and Development Program of Jiangsu Province (BE2022343).

## Author contributions

X.W., Y.X., and C.X. designed the research. X.W., Z.J., T.D., and Y.C. conducted the experiments. X.W., G.Y., and P.L. analyzed the data. X.W. wrote the paper. Z.Y.,S.X., Y.X., and C.X. revised the manuscript. All authors read and approved the final manuscript.

## Acknowledgments

No conflict of interest is declared.

## Supplemental information

Document S1. Figures S1 and S2 and Tables S1–S4

Document S2. Article plus supplemental information
