# Supplementary material for: GS-Impute: A neural network framework for accurate imputation of low-density markers in across-population genomic selection
Source: Plant Commun. 2026 Mar 10;7(5):101821. doi: 10.1016/j.xplc.2026.101821 (PMC13174246; doi:10.1016/j.xplc.2026.101821)
Supplement: Document S1. Figures S1 and S2 and Tables S1–S4 [file mmc1.pdf]

Plant Communications, Volume 7

Supplemental information

GS-Impute: A neural network framework for accurate imputation of low-density markers in across-population genomic selection

Xin Wang, Zhenting Jiang, Tongtong Ding, Ying Cao, Kai Zhou, Guangning Yu, Pengcheng Li, Zefeng Yang, Xuecai Zhang, Shizhong Xu, Yang Xu, and Chenwu Xu

Supplemental information

GS-Impute: a neural network framework for accurate imputation of low-density markers in across-population genomic selection

Xin Wang[1,2], Zhenting Jiang[1], Tongtong Ding[3], Ying Cao[1], Kai Zhou[2], Guangning Yu[2], Pengcheng Li[2], Zefeng Yang[2], Xuecai Zhang[4], Shizhong Xu[5], Yang Xu[2*], Chenwu Xu[2*]

[1] College of Information Engineering, Yangzhou University, Yangzhou 225009, Jiangsu, China/Jiangsu Engineering Research Center for Knowledge Management and Intelligent Service

[2] Key Laboratory of Plant Functional Genomics of the Ministry of Education/Jiangsu Key Laboratory of Crop Genomics and Molecular Breeding/Jiangsu Co-Innovation Center for Modern Production Technology of Grain Crops, College of Agriculture, Yangzhou University, Yangzhou 225009, Jiangsu, China

[3] Nantong Institute of Technology

[4] International Maize and Wheat Improvement Centre (CIMMYT), Texcoco, Me´xico

[5] Department of Botany and Plant Sciences, University of California, Riverside, CA, USA

*Corresponding authors: Chenwu Xu, E-mail address: cwxu@yzu.edu.cn; Yang Xu, E-mail address: yangx@yzu.edu.cn

Short Summary

GS-Impute is a neural network framework developed for accurate imputation of low-density markers in across-population genomic selection. Comprehensive evaluations in rice and maize showed that GS-Impute outperforms established benchmark tools, making low-density genomic selection a resource-efficient strategy for breeding programs.

Supplemental Figure legends

Supplemental Figure 1. Genotype r^2 (mean ± standard deviation across all chromosomes) with Tukey's HSD multiple comparison test results. For rice and maize, results from the 10 and 12 chromosomes are considered as independent samples, respectively. The corresponding mean values, standard deviations, and HSD test results are presented.

(A) Genotype r^2 for all loci in the testing set of 120 rice samples. **(B)** Genotype r^2 for all loci in the testing set of 100 maize samples.

Genotype r^2 is calculated as the squared correlation between imputed and true genotypes.

Supplemental Figure 2. Genotype r^2 across multiple minor allele frequency (MAF) intervals in the whole genome.

(A) Genotype r^2 for reconstructive imputation in the testing set of 120 rice samples. **(B)** Genotype r^2 for general imputation in in the testing set of 120 rice samples. **(C)** Genotype r^2 for reconstructive imputation in the testing set of 100 maize samples. **(D)** Genotype r^2 for general imputation in the testing set of 100 maize samples.

Genotype r^2 is calculated as the squared correlation between imputed and true genotypes.

Supplemental Table 1. Predictability of across-population GS for rice using different GS methods

Supplemental Table 2. Predictability of across-population GS for maize using different GS methods

Supplemental Table 3. Average predictability of within-population GS (GBLUP) for rice with SNP_{125K} via ten rounds of 5-fold cross-validation

Supplemental Table 4. Average predictability of within-population GS (GBLUP) for maize with SNP_{135K} via ten rounds of 5-fold cross-validation

Additional Supplemental Information

Pseudocode for the masked marker generation algorithm based on automatic matching of samples

Supplemental Figures

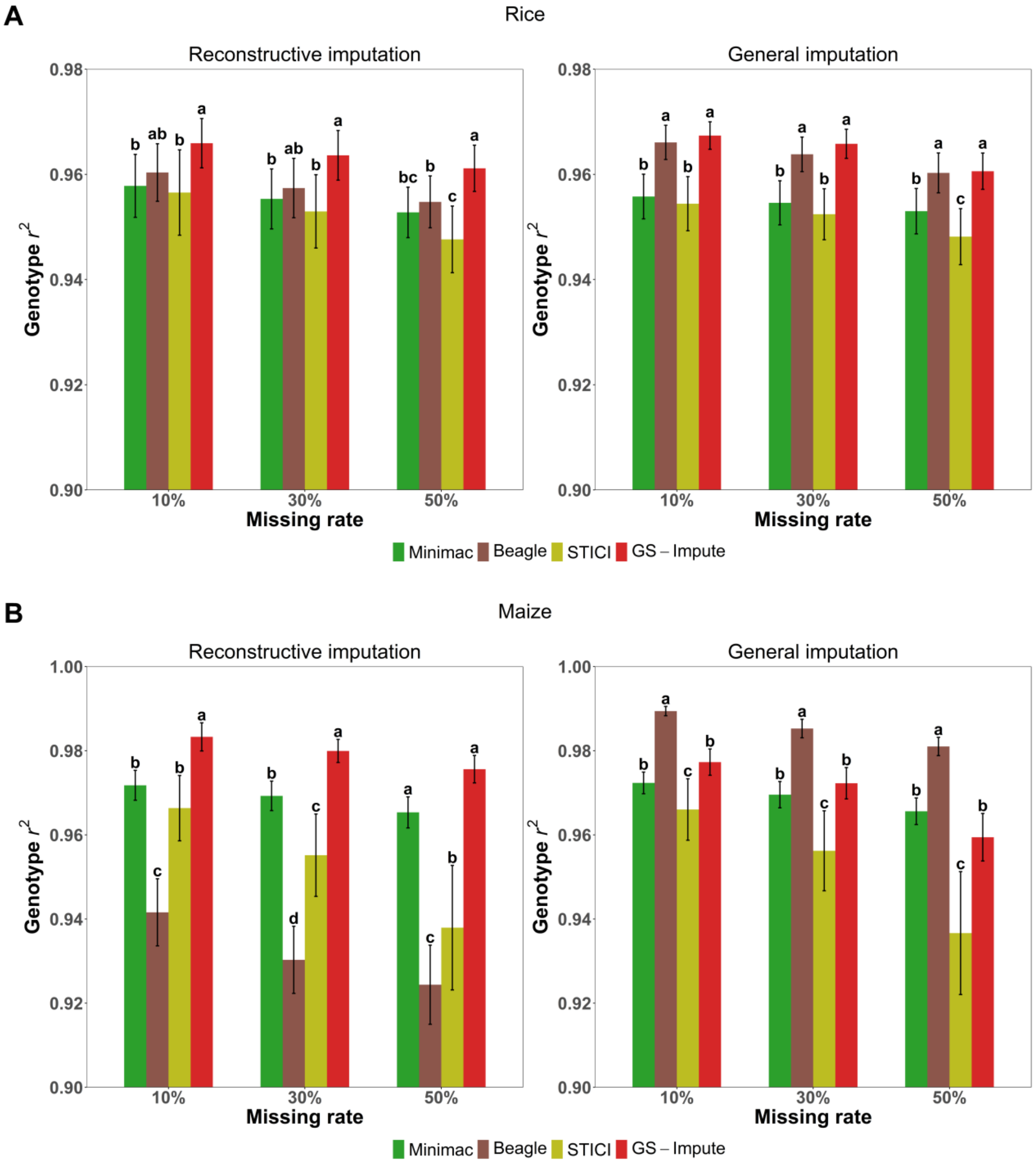

Supplemental Figure 1. Genotype r^2 (mean ± standard deviation across all chromosomes) with Tukey's HSD multiple comparison test results. For rice and maize, results from the 10 and 12 chromosomes are considered as independent samples, respectively. The corresponding mean values, standard deviations, and HSD test results are presented.

(A) Genotype r^2 for all loci in the testing set of 120 rice samples. **(B)** Genotype r^2 for all loci in the testing set of 100 maize samples.

Genotype r^2 is calculated as the squared correlation between imputed and true genotypes.

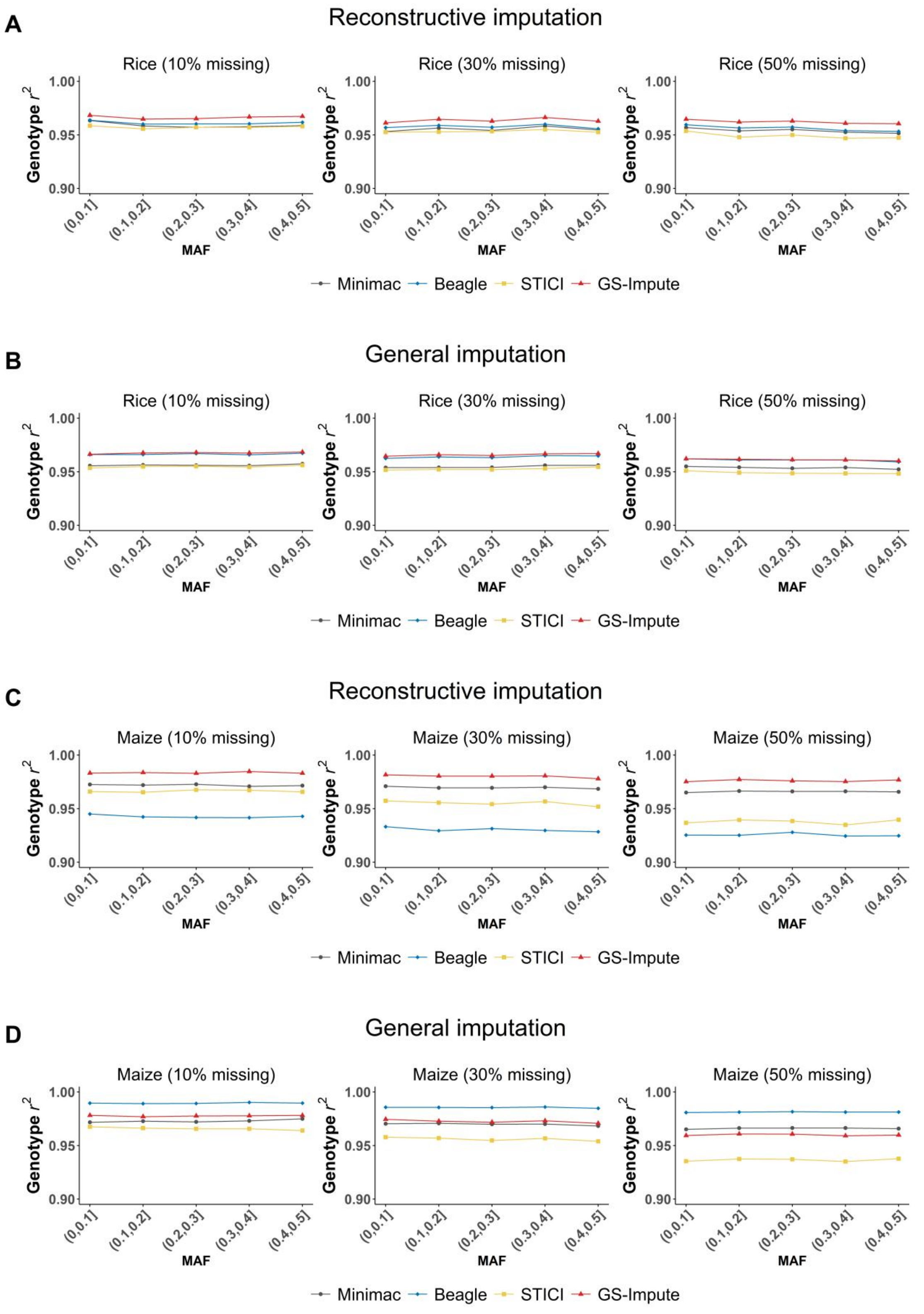

Supplemental Figure 2. Genotype r^2 across multiple minor allele frequency (MAF) intervals in the whole genome.

(A) Genotype r^2 for reconstructive imputation in the testing set of 120 rice samples. **(B)**

Genotype r^2 for general imputation in in the testing set of 120 rice samples. **(C)** Genotype r^2 for reconstructive imputation in the testing set of 100 maize samples. **(D)** Genotype r^2 for general imputation in the testing set of 100 maize samples.

Genotype r^2 is calculated as the squared correlation between imputed and true genotypes.

Supplemental Tables

Supplemental Table 1. Predictability of across-population GS for rice using different GS methods

GS scheme	Marker set	GS method	Trait			
			PN	GN	TGW	PL
Rice1495→Rice575	SNP_{125K}	GBLUP	0.238	0.345	0.758	0.580
		LASSO	0.201	0.250	0.784	0.565
		BayesB	0.244	0.349	0.762	0.585
	SNP_{113K}	GBLUP	0.241	0.352	0.756	0.582
		LASSO	0.188	0.240	0.769	0.563
		BayesB	0.243	0.311	0.754	0.585
Rice575→Rice1495	SNP_{125K}	GBLUP	0.248	0.299	0.663	0.159
		LASSO	0.111	0.174	0.597	0.201
		BayesB	0.222	0.309	0.666	0.096
	SNP_{113K}	GBLUP	0.247	0.286	0.662	0.160
		LASSO	0.132	0.220	0.564	0.069
		BayesB	0.254	0.249	0.639	0.123

Supplemental Table 2. Predictability of across-population GS for maize using different GS methods

GS scheme	Marker set	GS method	Trait					
			EW	ERN	KNR	ED	EL	PH
Maize633→Maize945	SNP_{286}	GBLUP	0.290	0.467	0.208	0.379	0.332	0.477
		LASSO	0.262	0.386	0.188	0.341	0.311	0.413
		BayesB	0.282	0.422	0.209	0.356	0.351	0.403
	SNP_{135K}	GBLUP	0.387	0.626	0.246	0.505	0.387	0.537
		LASSO	0.409	0.536	0.243	0.418	0.362	0.375
		BayesB	0.376	0.620	0.253	0.494	0.376	0.512
Maize945→Maize633	SNP_{286}	GBLUP	0.294	0.438	0.153	0.348	0.371	0.401
		LASSO	0.248	0.323	0.045	0.231	0.376	0.298
		BayesB	0.280	0.402	0.131	0.331	0.403	0.375
	SNP_{135K}	GBLUP	0.355	0.564	0.214	0.483	0.463	0.484
		LASSO	0.279	0.446	0.113	0.410	0.439	0.429
		BayesB	0.345	0.572	0.200	0.484	0.447	0.481

Supplemental Table 3. Average predictability of within-population GS (GBLUP) for rice with SNP_{125K} via ten rounds of 5-fold cross-validation

Data set	Trait			
	PN	GN	TGW	PL
Rice575	0.415	0.645	0.879	0.793
Rice1495	0.468	0.620	0.879	0.802

Supplemental Table 4. Average predictability of within-population GS (GBLUP) for maize with SNP_{135K} via ten rounds of 5-fold cross-validation

Data set	Trait					
	EW	ERN	KNR	ED	EL	PH
Maize945	0.677	0.796	0.482	0.766	0.565	0.777
Maize633	0.532	0.733	0.433	0.726	0.656	0.613

Additional Supplemental Information

Pseudocode for the masked marker generation algorithm based on automatic matching of samples

```
# Step 1: Input the numerically coded training set and the testing set containing missing values (0)
FUNCTION reconstructive_imputation_matching(train_x, testyin_x):
    train_num = LENGTH(train_x)
    # Step 2: Replace missing values in the testing samples with column means from the training samples
    meanList = MEAN(train_x, axis=0)
    testyin_x2 = COPY(testyin_x)
    FOR j FROM 0 TO LENGTH(testyin_x2[0]):
        missing_positions = FIND_INDICES_WHERE(testyin_x[:, j] == 0)
        testyin_x2[missing_positions, j] = meanList[j]
    # Step 3: Calculate the Manhattan distances between the testing samples and the training samples
    distArray = MANHATTAN_DISTANCES(testyin_x2, train_x)
    # Step 4: Calculate the average Manhattan distance between each testing sample and the training samples
    distMean = MEAN(distArray, axis=1)
    # Step 5: Sort the testing samples by distance (descending)
    argdistMean = SORT_INDICES_DESCENDING(distMean)
    # Step 6: Matching between the training and testing samples
    argTrain = EMPTY_LIST
    distArray = CONVERT_TO_DATAFRAME(distArray)
    WHILE LENGTH(argTrain) < train_num:
        FOR i IN argdistMean:
            remaining_cols = REMOVE_INDICES(distArray.iloc[i], argTrain)
            id_min = FIND_MIN_INDEX(remaining_cols)
            argTrain.APPEND(id_min)
            IF LENGTH(argTrain) == train_num:
                BREAK
    # Step 7: Generating the masked markers of the training samples
    k = 0
    train_x_corrupt = COPY(train_x)
    FOR i FROM 0 TO train_num-1:
        FOR j FROM 0 TO LENGTH(testyin_x[0])-1:
            IF testyin_x[argdistMean[k]][j] == 0:
                train_x_corrupt[argTrain[i], j] = 0
        k = (k + 1) MOD LENGTH(testyin_x)
    RETURN train_x, train_x_corrupt
```

```
# Step 1: Input the numerically coded training set and the testing set containing missing values (0)
FUNCTION general_imputation_matching(train_x, testyin_x):
    # Step 2: Self-imputation using KNN on the testing samples
    testyin_x0 = COPY(testyin_x)
    testyin_x3 = KNN_IMPUTER(testyin_x0)
    # Step 3: Replace missing values in the testing samples with column means from the training samples
    meanList = MEAN(train_x, axis=0)
    testyin_x2 = COPY(testyin_x)
    FOR j FROM 0 TO LENGTH(testyin_x2[0]):
        missing_indices = FIND_INDICES_WHERE(testyin_x2[:, j] == 0)
        testyin_x2[missing_indices, j] = meanList[j]
    # Step 4: Call the matching function of testing samples for augmentation
    masked_testing_results = test_matching_for_augmentation(testyin_x, testyin_x3)
    # Step 5: Calculate the average Manhattan distance between each testing sample and the training samples
    distArray = MANHATTAN_DISTANCES(testyin_x2, train_x)
    distMean = MEAN(distArray, axis=1)
    # Step 6: Sort the testing samples by distance (descending)
    argdistMean = SORT_INDICES_DESCENDING(distMean)
    # Step 7: Matching between the training and testing samples
    argTrain = EMPTY_LIST
    distArray = CONVERT_TO_DATAFRAME(distArray)
    WHILE LENGTH(argTrain) < train_num:
        FOR i IN argdistMean:
            remaining_cols = REMOVE_INDICES(distArray.iloc[i], argTrain)
            id_min = FIND_MIN_INDEX(remaining_cols)
            argTrain.APPEND(id_min)
            IF LENGTH(argTrain) == train_num:
                BREAK
    # Step 8: Generating the masked markers of the training samples
    k = 0
    train_x_corrupt = COPY(train_x)
    FOR i FROM 0 TO train_num-1:
        FOR j FROM 0 TO LENGTH(testyin_x[0])-1:
            IF testyin_x[argdistMean[k]][j] == 0:
                train_x_corrupt[argTrain[i], j] = 0
        k = (k + 1) MOD LENGTH(testyin_x)
    # Step 9: Stack the training set with the masked testing set to obtain the augmented training set
    train_x = VERTICAL_STACK(train_x, masked_testing_results[0])
    train_x_corrupt = VERTICAL_STACK(train_x_corrupt, masked_testing_results[1])
RETURN train_x, train_x_corrupt
```

```
FUNCTION test_matching_for_augmentation(testyin_x, testyin_x_knn):
    train_num = LENGTH(testyin_x)
    # Step 1: Calculate the Manhattan distances between KNN-imputed samples
    distArray = MANHATTAN_DISTANCES(testyin_x_knn, testyin_x_knn)
    SET_DIAGONAL(distArray, MAX(distArray) * 2)
    # Step 2: Calculate the average distances and sort indices
    distMean = MEAN(distArray, axis=1)
    argdistMean_desc = SORT_INDICES_DESCENDING(distMean)
    argdistMean_asc = SORT_INDICES_ASCENDING(distMean)
    # Step 3: Matching with two selection strategies (descending and ascending)
    argTrain_desc = EMPTY_LIST
    argTrain_asc = EMPTY_LIST
    distArray_df = CONVERT_TO_DATAFRAME(distArray)
    train_num_selected = train_num
    # Strategy 1: Matching based on descending distance order
    WHILE LENGTH(argTrain_desc) < train_num_selected:
        FOR i IN argdistMean_desc:
            remaining_cols = REMOVE_INDICES(distArray_df.iloc[i], argTrain_desc)
            id_min = FIND_MIN_INDEX(remaining_cols)
            argTrain_desc.APPEND(id_min)
            IF LENGTH(argTrain_desc) == train_num_selected:
                BREAK
    # Strategy 2: Matching based on ascending distance order
    WHILE LENGTH(argTrain_asc) < train_num_selected:
        FOR i IN argdistMean_asc:
            remaining_cols = REMOVE_INDICES(distArray_df.iloc[i], argTrain_asc)
            id_min = FIND_MIN_INDEX(remaining_cols)
            argTrain_asc.APPEND(id_min)
            IF LENGTH(argTrain_asc) == train_num_selected:
                BREAK
    # Step 4: Merge the selected indices from both strategies and sort
    selected_indices = UNION(argTrain_desc, argTrain_asc)
    selected_indices = SORT(selected_indices)
    # Step 5: Generating the masked markers of the testing samples for training
    testyin_x2 = COPY(testyin_x_knn)
    FOR i FROM 0 TO LENGTH(argTrain_desc)-1:
        FOR j FROM 0 TO LENGTH(testyin_x[0])-1:
            IF testyin_x[argdistMean_desc[i]][j] == 0 AND testyin_x[argTrain_desc[i]][j] != 0:
                testyin_x2[argTrain_desc[i]][j] = 0
            IF testyin_x[argdistMean_asc[i]][j] == 0 AND testyin_x[argTrain_asc[i]][j] != 0:
                testyin_x2[argTrain_asc[i]][j] = 0
    testyin_x_original = testyin_x_knn[selected_indices, :]
```

```
    testyin_x_corrupted = testyin_x2[selected_indices, :]
RETURN testyin_x_original, testyin_x_corrupted
```
